# Epidemiological investigation of an outbreak of cutaneous sporotrichosis, Northern Territory, Australia

**DOI:** 10.1186/s12879-016-1338-0

**Published:** 2016-01-13

**Authors:** Sarah L. McGuinness, Rowena Boyd, Sarah Kidd, Charlie McLeod, Vicki L. Krause, Anna P. Ralph

**Affiliations:** 1 Royal Darwin Hospital, Rocklands Drive, Tiwi, Darwin, NT 0810 Australia; 2 Centre for Disease Control, Department of Health, Royal Darwin Hospital Campus, Rocklands Drive, Tiwi, Darwin, NT 0810 Australia; 3 National Mycology Reference Centre, Microbiology & Infectious Diseases, SA Pathology, Frome Road, Adelaide, SA 5000 Australia; 4 Global and Tropical Health, Menzies School of Health Research, Royal Darwin Hospital Campus, Rocklands Drive, Tiwi, Darwin, NT 0810 Australia

**Keywords:** Sporotrichosis, Sporothrix, Disease outbreaks, Epidemiology, Itraconazole, Adult, Humans, Australia

## Abstract

**Background:**

An outbreak of cutaneous sporotrichosis occurred in the Darwin region of the Northern Territory (NT) in 2014. We aimed to determine the source and risk factors associated with the outbreak and describe the clinical spectrum of cases seen.

**Methods:**

Epidemiological investigation of cases of cutaneous sporotrichosis identified through the Royal Darwin Hospital was undertaken to investigate risk factors and potential sources of infection. Data were collected through chart review and individual patient interviews. Environmental investigation followed identification of a common risk factor.

**Results:**

Nine confirmed cases of cutaneous sporotrichosis caused by *Sporothrix schenckii* were identified with onset of symptoms between April and July 2014. Patients were aged 29 to 70 years and seven were male (78 %). Two strains of *S. schenckii* were identified, neither of which have been previously documented. One common risk factor was identified: all patients were occupational or recreational gardeners, with each reporting exposure to mulching hay, originating from a single NT farm. Local environmental health officers visited the farm and the owners confirmed that the implicated hay had been stored over the monsoon season and had been affected by rain. Storage of hay over the wet season was a new practice.

**Conclusions:**

This constitutes the third reported outbreak of *S. schenckii* sporotrichosis attributable to contaminated hay in Australia and the first outbreak of sporotrichosis in the NT. This outbreak prompted public health interventions, including distribution of information to general practitioners, farmers and suppliers in the Top End. Media reporting led to the identification and treatment of an additional case. Local practitioners should remain alert to the possibility of further occurrences of sporotrichosis.

## Background

Sporotrichosis is an infection caused by dimorphic fungi in the genus *Sporothrix*. These are environmental saprophytes associated preferentially with decaying vegetation and soil [1]. Human infection can occur sporadically or in outbreaks. Infection generally follows traumatic cutaneous inoculation of matter contaminated with the fungus [2]. Cases of animal-to-human transmission have also been described [3, 4]. Most clinical cases are limited to the skin, subcutaneous tissue and adjacent lymphatics; dissemination to other organs is rare [1].

*Sporothrix* species*,* in particular *S. schenckii,* are recognised to have a near-worldwide distribution including in Australia, with higher prevalence in tropical and subtropical zones where high temperature and humidity favour growth of the organism [5]. *Sporothrix brasiliensis* and *Sporothrix mexicana* are noted to have regional foci in South America, *Sporothrix globosa* in Europe and East Asia and *Sporothrix luriei* has been rarely isolated in South Africa [6]. Three *Sporothrix* species–*S. schenckii*, *S. globosa* and *S. mexicana*–have been documented in Australia [7, 8].

Darwin is located in the ‘Top End’ of Australia and has a tropical climate with an annual monsoon season (November to April). Sporadically occurring cases of sporotrichosis have been noted to occur in the NT in the past, but two cases of sporotrichosis presenting in 2013 were the first NT acquired cases to be published in the literature [9]. Both affected individuals described multiple potential inoculating injuries, and hence a common source could not be identified at that time. We describe the clinical characteristics and risk factors associated with nine cases of sporotrichosis occurring in 2014 in patients from the Darwin region, and present the findings of the ensuing public health investigation.

## Methods

Formal ethics committee approval was not required as this study was part of an outbreak investigation by the Centre for Disease Control, Darwin. However, verbal informed consent was obtained from all participants for use and publication of their clinical details and written informed consent for use of clinical photography images. Active case surveillance was initiated by the infectious diseases and microbiology departments at the Royal Darwin Hospital in August 2014 after several cases of sporotrichosis were identified. Using the microbiological databases of the two major pathology laboratories servicing the NT, a search was conducted to identify all *S. schenckii* isolates from the previous 10 years (2004–2014) to estimate the background rate of infection and identify potentially related cases.

A case of sporotrichosis was defined as clinical evidence of disease supported by microbiological isolation of *S. schenckii* from culture of tissue specimens and/or identification of yeasts consistent with *S. schenckii* on histopathological examination of tissue specimens in any person residing in or visiting the Darwin region of the NT. Microbiological identification was initially performed by morphologic and phenotypic methods, and later confirmed by DNA sequencing of the ribosomal DNA Internal Transcribed Spacer 1 and 2 regions (ITS). ITS sequences were compared against the NCBI and CBS sequence databases, which included recently published ITS sequences of all known *Sporothrix* species [6]. Antifungal susceptibilities were determined according to the CLSI M38-A2 standard on the mycelial growth phase.

Clinical data were collected through chart review of clinical and microbiological databases. Data collected on cases included demographic characteristics, duration of symptoms, clinical findings, laboratory investigations, treatment, and response to therapy. Epidemiological data were collected through individual telephone interviews exploring demographic factors and potential risk factors for exposure to *S. schenckii*. Data collected included occupation, relevant hobbies, and exposure to organic matter, mine sites, pets and animals.

Environmental investigation followed identification of a common risk factor. Interview of the farm manager established hay production, storage and distribution practices. An inspection of hay storage sheds was undertaken.

## Results

### Microbiological database search

Between 2004 and 2014, 13 clinical isolates of *S. schenckii* were identified by the two laboratories: 8 in 2014, 3 in 2013, 1 in 2012 and 1 in 2010. No isolates were identified between 2004 and 2009 at either laboratory. Detailed clinical information was available for all 8 isolates identified in 2014 (two of which were from the same patient) and are described here. Clinical information about the 2013 cases has been described elsewhere [9]. These comprised two NT acquired cases and one case acquired in Queensland. No clinical information was available for the isolates from 2012 and 2010.

### Clinical and demographic characteristics

**Fig. 1:**
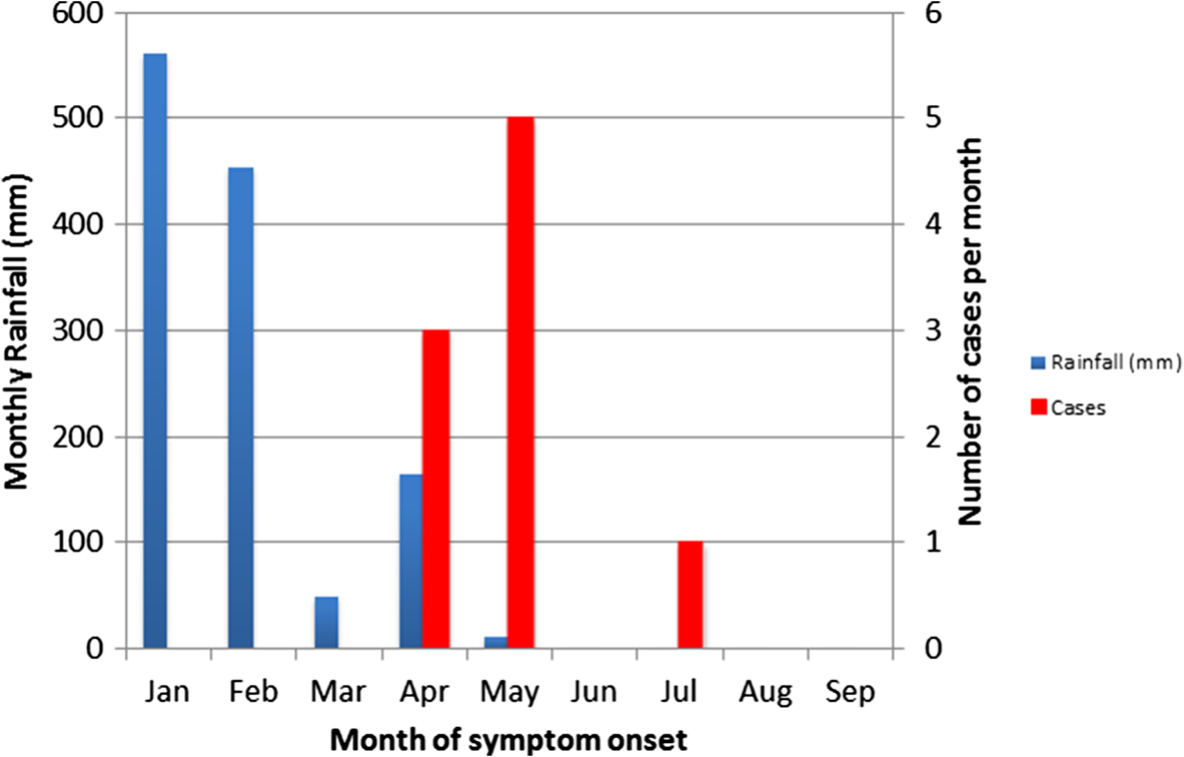
Rainfall data and epidemic curve of sporotrichosis cases by month of symptom onset, Darwin region 2014 [14]

**Fig. 2:**
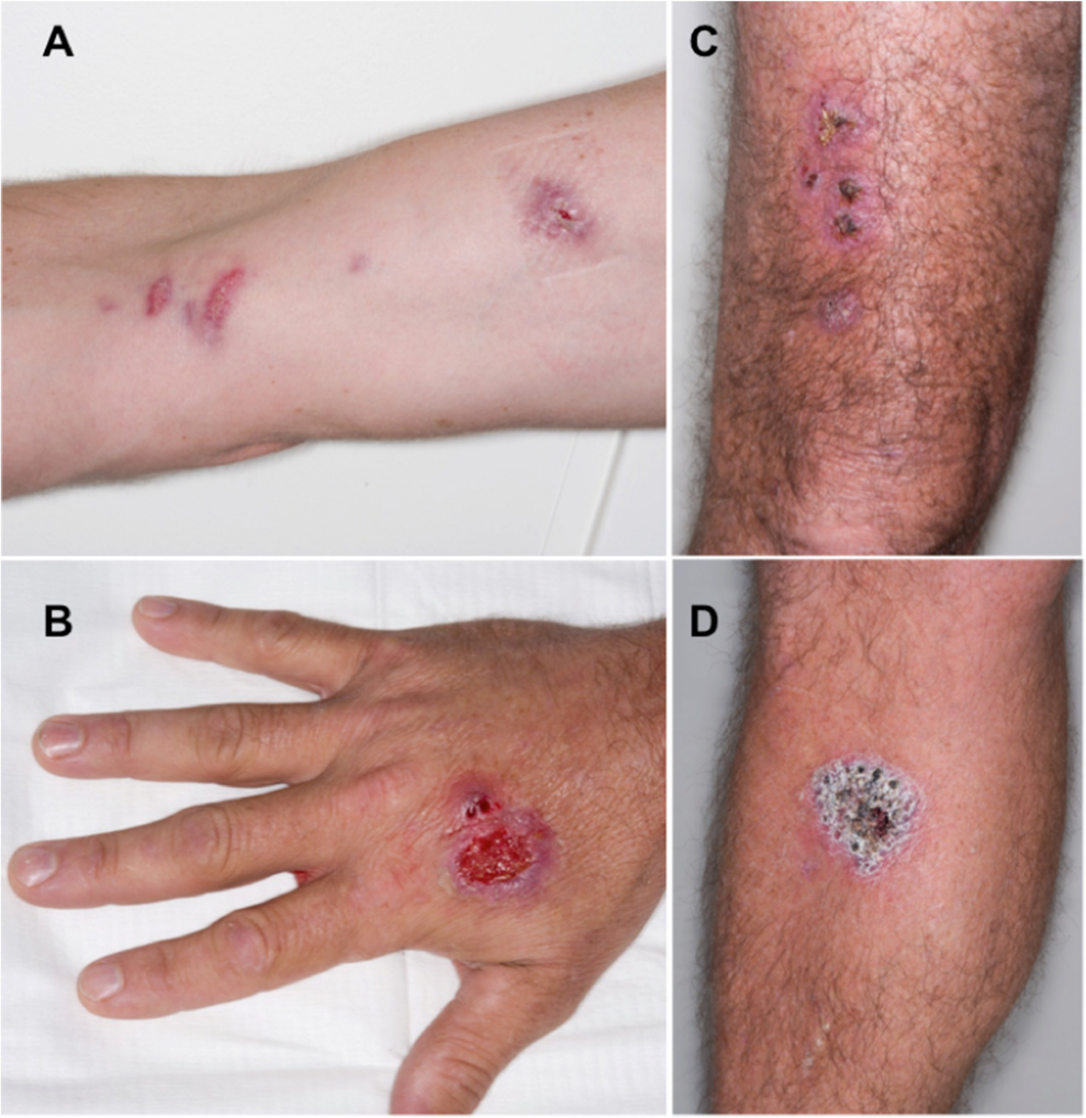
Cases of sporotrichosis. **a**: lymphocutaneous spread from cubital fossa lesion (case 7). **b**: primary ulcerated lesion on right hand, with adjacent satellite nodule from which two 3 mm punch biopsies have been taken (case 5). **c**: fixed cutaneous lesions on right thigh–patient recalled multiple scratches sustained when unloading hay bales from his utility truck (case 8). **d**: single crusted lesion on right lower leg (case 6)

Between July 2014 and March 2015, nine confirmed cases of sporotrichosis were diagnosed in NT residents (Table 1 and Fig. 1). Patients were aged 29 to 70 years (mean age 47) and seven were male (78 %). None were immunocompromised or had other known risk factors, although one male reported hazardous alcohol consumption (6–12 standard drinks daily). Seven patients had lesions involving the upper limb and four had lesions involving the lower limb. Eight patients had multiple lesions. Lymphocutaneous sporotrichosis (sporotrichoid spread) was observed in six patients (67 %); the others had fixed cutaneous disease (Fig. 2). No patient had evidence of extracutaneous or disseminated disease. The earliest illness onset was in April 2014, corresponding with the end of the wet season, and the last in July 2014. The time to diagnosis from onset of first symptoms ranged from one to eleven months.

**Table 1:** Sporotrichosis clinical presentation, diagnosis and treatment

Case	Other medical problems	Month of symptom onset	Time to diagnosis (months)^a^	Site of primary lesion	Sporotrichoid spread	Multiple lesions	Culture confirmed	Sequence type (ST); Genbank accession	Itraconazole dose prescribed	Treatment duration (months)
1	Hypertension	May	2	Lower leg (R)	Yes	Yes	Yes	ST 1; KU041841	200 mg daily	3
2	N/A	May	2	Multiple (both arms, chest, left leg)	No	Yes	Yes	ST 2; KU097326	200 mg daily	4
3	N/A	May	2	Forearm (R)	Yes	Yes	Yes	ST 1; KU041840	200 mg daily	6
4	N/A	May	2	Finger (R)	Yes	Yes	No^b^	N/A	100 mg daily	3
5	Smoker	July	1	Hand (R)	Yes	Yes	Yes	ST 2; KU041843	200 mg daily	3
6	Ischaemic heart disease, hazardous alcohol	May	3	Lower leg (R)	No	No	Yes	ST 2; KU041846	200 mg daily	3
7	N/A	April	4	Cubital fossa (R)	Yes	Yes	Yes^c^	N/A	200 mg daily	6
8	Hypertension	April	5	Thigh (R)	No	Yes	Yes	ST 2; KU041845	200 mg daily	4
9	N/A	April	11	Forearm (L)	Yes	Yes	Yes	ST 1; KU041842	200 mg daily	Ongoing

^a^Time from onset of symptoms to confirmation of diagnosis (by culture or histopathology)

^b^Fungal culture not performed. Diagnosis based on characteristic histopathology findings and consistent clinical and epidemiological features

^c^Isolate could not be revived for ITS sequencing following its morphological identification as *S. schenckii* complex

Presumptive *S. schenckii* was isolated from tissue biopsy in eight cases. One patient (case 7) also had *S. schenckii* isolated from a wound swab. The average lag time from time of biopsy to culture confirmation was 4 weeks. Identification was confirmed as *S. schenckii sensu stricto* by ITS sequencing in 7 of 8 culture confirmed cases; the morphological identification could not be confirmed by ITS sequencing for case 7 as the stored isolate could not be revived. Minimum inhibitory concentrations (MIC) to itraconazole ranged from 0.5 to 1.0 μg/mL (mode 1.0 μg/mL).

The 7 sequenced isolates comprised two ITS sequence types, designated here as ST 1 and ST 2, differing by a single nucleotide (378, ITS2 region). The isolates from two previously described 2013 NT acquired cases [9] were also sequenced and were found to possess the same two ITS sequence types (Genbank accessions KU041839, KU041844) identified in this 2014 case series. All ITS sequences from the NT cases differed by 1–2 nucleotides from any previously published sequences.

Yeast forms were seen on histopathology in seven of the nine cases. The only non culture-confirmed case (case 4) had characteristic histopathological findings, with suppurative granulomatous inflammation and yeast forms on periodic acid-Schiff stain, and had been commenced on empiric itraconazole by her general practitioner based on these results prior to referral to the infectious diseases clinic so fungal culture was not performed.

All patients responded well to oral itraconazole treatment at a dose of 100–200 mg daily. Therapeutic monitoring of itraconazole levels was undertaken in 3 patients, according to clinician preference. Dosage change in response to therapeutic monitoring occurred in one patient; itraconazole dosage was increased from 200 mg daily to 200 mg twice daily after a reported trough concentration of 883 μg/ml, but a repeat itraconazole level of 2527 μg/ml necessitated a return to the original 200 mg daily dose. At the time of this report, all but one patient had completed their planned treatment course. One patient experienced a relapse, comprising enlargement of a tiny residual lesion and new satellite lesion, after having completed itraconazole 100 mg daily for 3 months with good adherence, and was recommenced on therapy at a dose of 200 mg daily with a good response.

### Epidemiological characteristics

The nine patients identified had different occupations in different workplaces. All lived within a 60 km radius of Darwin: one in urban Darwin, five in the outer suburban areas of Darwin and Palmerston, and three in the Litchfield region. All patients were occupational or recreational gardeners and had handled bales of mulching hay, purchased from one of two hay suppliers. Other potential exposures included domestic animals or pets 8/9 (89 %), predominantly dogs 6/9 (67 %) and/or chickens 4/9 (45 %). In the 3 months preceding onset of symptoms 6/9 (67 %) patients reported contact with commercially sold potting mix, 3/9 (33 %) visited a farm, 3/9 (33 %) had contact with moss and 1/9 (11 %) had visited a mine site.

All nine patients purchased hay between December 2013 and May 2014. While five patients purchased hay directly from a local farm and four patients purchased hay from a local nursery, all purchased hay was identified to have originated from a single hay supplier. One patient bought and handled hay in February 2014, seven patients bought and handled hay in April 2014, and the final patient bought hay on multiple occasions between December 2013 and May 2014 and spread hay on a fortnightly basis in his chicken coop. Only one patient reported wearing protective clothing while handling hay. Three patients reported the hay appeared mouldy as evidenced by white patches or dark, damp areas in the middle of the bale.

### Environmental investigation

Local environmental health officers visited the hay supplier from which all purchased hay had originated. The owners of the farm confirmed that over the past two monsoon seasons (2012–2013 and 2013–2014), hay had been stored in a semi-enclosed shed and had been affected by rain. This would have provided conditions appropriate for the growth of *S. schenckii* within hay bales. Storage of hay over the monsoon season was a new practice; prior to the 2012–2013 wet season, all hay had been sold during the dry season. Mycological culture of hay samples was not performed, but the epidemiological link and biological plausibility were considered persuasive.

### Public health response

Information regarding the occurrence of sporotrichosis following exposure to hay was distributed to general practitioners, hay manufacturers and suppliers in the district. Recommendations made to producers and suppliers included to: (i) avoid storing hay during the monsoon season; (ii) avoid sale of hay bales with obvious mould contamination on visual inspection and (iii) distribute information leaflets with hay sales, advising people to wear protective clothing (e.g. gloves and long sleeved shirts) when handling hay. A media release issued by the Northern Territory Centre for Disease Control led to outbreak coverage in local print media and radio. This resulted in the identification and treatment of case 8, who contacted the public health unit after seeing the story in the local newspaper.

## Discussion

This constitutes the third reported outbreak of sporotrichosis attributable to contaminated hay in Australia, with previous outbreaks occurring in Western Australia and Queensland [8, 10]. An epidemiological investigation implicated mulching hay sourced from a single farm. All patients who developed sporotrichosis were occupational or recreational gardeners, with each reporting exposure to mulching hay during the early dry season after the monsoon. In retrospect, it is likely that exposure to contaminated hay was the cause of the two locally acquired sporotrichosis cases diagnosed in 2013; both reported contact with mulching hay (although only one was able to confirm purchase from the implicated supplier) and sequencing of these isolates demonstrated the same two ITS sequence types identified from the 2014 cases. The occurrence of an outbreak of sporotrichosis in this geographical setting correlated with the storage of hay in an exposed area during the monsoon season, a change from previous farming practice. An association between sporotrichosis and rainfall has also been postulated in another Australian setting [11].

All seven isolates from this case series for which ITS sequences were obtained, along with those from two 2013 NT cases [9], possessed one of two ITS sequence types that have not previously been documented or represented in publically accessible sequence databases. This is suggestive of strains that may be unique to the NT or Australia, and supports the hypothesis of common source(s) of infection. These unique sequences were submitted to Genbank (accession numbers listed in Table 1).

While the clinical and epidemiological data gathered during this outbreak investigation provides strong circumstantial evidence of a link between sporotrichosis cases and mouldy hay, mycological culture of hay samples was not performed and thus we are unable to provide conclusive evidence that hay was the source of infection.

Sporotrichosis remains uncommon in Australia; 199 cases have been reported in the literature, but the disease is not notifiable, so the true incidence is unknown [9]. Data acquired from two NT laboratories suggests that prior to 2013, cases of sporotrichosis have been infrequently diagnosed. Failure to recognise sporotrichosis and perform the requisite biopsy for fungal culture and/or histopathological examination is common, and frequently results in prolonged diagnostic delays (ranging 1–11 months in our series), and unnecessary antibiotic use. Many of the patients reported here were treated with multiple courses of antibiotics before a biopsy was performed. In tropical locations, the list of differential diagnoses for chronic skin lesions with or without sporotrichoid spread is long, presenting particular challenges for diagnosis [9, 12]. A practice point learned from this case series is the importance of continuing treatment for 2–4 weeks beyond complete resolution of lesions to prevent relapse, as recommended in practice guidelines [13]. Therapeutic drug monitoring (TDM) of itraconazole trough concentrations did not appear to be helpful in this series, given that the only TDM-prompted dose change needed to be changed back again after levels subsequently exceeded the recommended range. In general, TDM of itraconazole trough concentrations is recommended in patients with visceral involvement with sporotrichosis and those with lymphocutaneous disease who are experiencing failure of therapy [13].

## Conclusions

This comprises the first reported outbreak of sporotrichosis in the NT and the third reported outbreak of *S. schenckii* sporotrichosis attributable to contaminated hay in Australia. This outbreak prompted public health interventions, including distribution of information to general practitioners, farmers and hay suppliers in the NT. Media reporting led to the identification and treatment of an additional case. Although our recommendations included avoidance of hay storage over the monsoon season and discarding bales with obvious mould contamination, these practices are difficult to monitor. Given the lack of obvious mould contamination reported in some of the implicated bales and a general reluctance to wear long clothing in an area of high ambient temperature and humidity; local practitioners should remain alert to the possibility of further occurrences of sporotrichosis.

## Abbreviations

MIC: minimum inhibitory concentration
NT: Northern Territory
TDM: therapeutic drug monitoring
ITS: internal transcribed spacer of the ribosomal DNA
ST: Sequence type
